# Practical Treatise on Diseases of the Eye

**Published:** 1835-01-01

**Authors:** 


*Practical Treatise on Diseases of the Eye.* By WM. MACKENZIE, M.D. *Second Edition.* London, 1834. 8vo.

IT is somewhat more than four years since this work was presented to the profession; a second edition, in a new and more extended form, has now given us a double opportunity of congratulation,—to the author, on his well-earned reputation and success,—to our medical brethren, on the stand which ophthalmic medicine and surgery has of late taken in the ranks of British science.

It is needless to enter into a history of this subject, since, in the first number of our Journal, the question was discussed, in the review of Mr. Lawrence's treatise; yet we may remark that, considering the perfect organization of the eye, and the high intrinsic interest of the maladies to which it is liable, it is somewhat singular that the morbid conditions of this organ should not be made still further available to the general elucidation of pathology and therapeutics.

In the eye, and its adjuncts, we meet with almost, nay all, the tissues of which the organism is compounded, all the varieties of disease, all accidents to which these textures are exposed. Here we trace the commencement of inflammation, and its progress through all its phases, to destruction of the organ, or to cure. We have the effects of our remedial agents, and in some instances even the *modus operandi* of our plan, "oculis subjecta fidelibus." We see the tubercle of lymph, in iritis, eaten into as it were day by day, under the sorbefacient influence of mercury; the scattered fragments of a cataract disappearing, by the solvent action of the aqueous fluid; or the ulcer healing, as the inflammatory symptoms abate, and the constitution regains its tone. After all this field of observation, shall we not be guided by those general principles which are demonstrated in the eye, in our application to the more obscure diseases of the concealed viscera? It is not for the eye alone that the investigation of ophthalmic pathology is so highly interesting, but, as in other sciences, we learn the unknown by the known, and the unseen changes which take place in the liver or the heart may be more than guessed at, from what we have observed in the eye.

In an organ which differs so little in actual magnitude, (for, what is called *a small eye*, often depends only upon the size of the palpebral fissure,) we think that the exact admeasurements, as given by our author, and the reference to a scale of Parisian lines with the division of our own inch (vide Plate 2, p. xxxiv.) is a circumstance of no little importance, especially as relates to some of the more delicate manipulations of the surgeon.

In speaking of the sclerotic coat, at p. xxii. reference is made to a serous membrane, more particularly described when mentioning the attachments of the choroid to the sclerotic: thus we find,

"This serous membrane, called *arachnoidea oculi*, is reflected from the sclerotica to the choroid, posteriorly, around the entrance of the optic nerve; anteriorly, behind the ciliary ligament; and at every place where a vessel or nerve passes from either of these tunics to the other. There being a great many of such vessels and nerves, especially at the back part of the eye, the cavity of the arachnoidea oculi is rendered less evident there than anteriorly." (P. xxiv.)

Now we remember to have read in some old authors how the tunics of the eye were expansions of the membranes of the brain, blown out as it were like a globule of melted glass from the end of a tube. Thus the dura mater gives us the sclerotic, the tunica arachnoides the membrane in question, and the pia mater the choroid, while the actual substance of the brain is to be found in the retina. We suspect, however, notwithstanding the elegance of this theory, that the "arachnoides oculi" is only a fine cellular membrane of connexion; and this is further confirmed by the examination of the fœtal eye, which, if placed in water, shows many fine flocculi adherent to the sclerotic and choroid coats, not dependent alone upon the ruptured extremities of nerves and vessels, as may more clearly be distinguished if the organ be previously injected with coloured size. That a serous membrane exists, however, adherent on the one part to the choroid, we do indeed believe; but then this is on the internal surface of the tunic, and is the membrane of the pigment on the one hand, and the tunica Jacobi on the other. That this choroidal reflexion does not secrete the pigment, is proved by its existence in the albino; and that it terminates at the *ora serrata,* is, we think, well marked by the defined scalloped border of a more intensely coloured pigment. This colouring matter is, we apprehend, secreted *behind* the serous covering as regards the choroid; *anterior* to the zonula Zinnii in reference to the corpus ciliare; and *behind* the delicate membrane which lines the posterior chamber, as regards the iris or uvea, (*vide* p. xxvi and vii.)

The following is the account here given of that very interesting point, the so-called " foramen of Sömmering:"

" In the direction of the axis of the eye, the retina is raised into a small fold, which extends from near the temporal side of the entrance of the optic nerve transversely outwards for about two lines. Here the retina presents a spot, about two lines in diameter, of a fine yellow colour, deeper in tint at the centre than at the circumference, and about the middle of the spot, in a transparent point. These parts were discovered, in 1791, by S. T. Sömmering, who, considering the transparent point a hole, called it *foramen retinæ centrale*, and the yellow spot *limbus luteus foraminis centralis.* The appearances just described are found only in the human eye, and in that of quadrumanous animals. In the chameleon and some other lizards, there is a central point or hole, which appears continuous with the small fissure through which the rudimental pecten of those animals projects, a circumstance which goes to support the hypothesis that there is an analogy between the central point of the retina, and the fissure in the retina of birds, for the passage of the pecten. This hypothesis, first suggested by Professor Huschke of Jena, has its principal foundation in the appearances presented by the eye in the different stages of its developement, but into which it would be foreign to our purpose to enter. There have been many opinions regarding the nature of the yellow spot. Dr. Ammon of Dresden, one of the latest writers on the subject, is of opinion, and in this he is joined by Dr. Arnold, that the yellow spot is owing to the black pigment changed in colour. That the pigment is there sometimes of a yellowish brown colour is true, but its tint is very different from that of the yellow spot; moreover, the pigment is separated from the yellow spot by the interposition of the membrane of Jacob. From examination with the microscope, it appears that the yellow spot of the retina is owing to yellow globules. In birds, the whole of the outer surface of the retina is covered by a layer consisting of yellow globules, the tint of which is deeper around the fissure of the retina which gives passage to the pecten. May not, then, the yellow spot of the human retina be a rudiment of that layer of yellow matter found on the outside of the retina of birds? This supposed analogy corresponds with that which has been mentioned to exist between the central point of the human retina and the fissure in that of the bird, for the passage of the pecten. The analogue of the central fold of the human retina may also be found in that which the retina of the bird presents at an early period in connexion with the pecten." (P. xxviii.)

In the eye of a chameleon now before us, the central point or hole is at the distance of a line and half from the pecten, and there is no *fissure* continuous with it, but a double fold, which appears analogous to the fold of the human retina, in the centre of which the yellow spot is generally seen. In the eye of the human fœtus at the term, in which the fold is often very visible, and in two instances which we have attentively examined, the aperture penetrates the retina, properly so called, but not the tunica Jacobi. These preparations underwent no manipulation beyond the removal of a portion of the choroid and tunic of Jacob from the back of the eye; a transverse section being then made just behind the cornea, the vitreous body and lens fell out, leaving the fold and foramen perfectly distinct; a free ray of light now penetrates this aperture, exhibiting the reality of the perforation.

Although our author's anatomical introduction is very short, yet it is well put together, and contains much of what is yet but little known, and for which we are indebted to the minute labours of the German anatomists. Some of the more delicate structures, however, require farther examination, and the descriptions given should be verified by other observers.

In a work containing upwards of one thousand pages, the choice of subjects for comment becomes difficult, from their very abundance. Upon mature consideration, we think that the portion of the work dedicated to the examination of the more common maladies of the eye, is, for many reasons, the most deserving of our attentive study: principally, indeed, because the various permanent defects of this organ, as the results of disease, are all, more or less, dependent upon previous inflammatory action of some or other tunic. The treatment, moreover, of the different forms of inflammation, originating from specific causes, or modified by variable conditions of constitution, affords us a fair specimen of our author's powers of investigation, and of the talent and skill which he brings to bear upon his subject.

Dr. Mackenzie enters upon his account of ophthalmiæ in general, by mentioning the primary symptoms of inflammation, as "increased redness, unnatural heat, swelling, and pain;" and the secondary train, as "effusion of red blood, colourless blood or fibrine, adhesion, suppuration, ulceration, mortification, granulation, and cicatrization." After these come the tertiary set of inflammatory phenomena, dependent upon the secondary, viz. "hypertrophy, atrophy, induration, softening, &c." From these data he proceeds:

"Inflammation, in whatever part of the body, and consequently in whatever part of the eye, it exists, may terminate in any of the processes now enumerated. It is also well known that the secondary and tertiary phenomena of inflammation are always modified, according to the structure of the part affected. Every different texture of the eye, as it possesses both physical and vital properties peculiar to itself, must suffer differently from these several processes of inflammation. In general, the modifications of inflammation, from differences of textures in the parts affected, are displayed with much distinctness in this organ: in some cases these modifications can be judged of only from their consequences, and by a very minute observation of the derangement which remains in the organization or in the function of the part which had suffered; while, in other cases, from the delicate texture of the part, or its hidden situation in the eye, the modifications in question may altogether escape observation.

"The conjunctiva, sclerotica, cornea, and iris, present a series of the modifications of inflammation to which I have just now referred, sufficiently distinct to convince the most sceptical of the truth of what I have been asserting, and sufficiently striking to rouse the most inattentive to research. The muco-cutaneous conjunctiva secreting a flood of purulent matter, as in the contagious ophthalmiæ,—the fibrous sclerotica affected for months with rheumatic inflammation,—the cornea losing entirely its transparency, becoming infiltrated with pus, or destroyed layer after layer by a penetrating ulcer,—the iris pouring out coagulable lymph, and this lymph forming the medium of morbid adhesions, so that the pupil is deprived of its natural power of expanding and contracting: these are facts in which are displayed some of the modifications of inflammatory action, more distinctly and strikingly than they are manifested in any other part of the body.

"There are other circumstances besides differences of texture which modify the inflammatory affections of the eye, rendering also this subject very extensive in the discussion, and causing the diseases to be occasionally very perplexing in the treatment. They are under the influence of peculiarities of constitution, and of constitutional diseases, and are subject to innumerable variations from the influence of sympathies. Scrofula, syphilis, and gout, are each of them capable either of exciting inflammation in different parts of the eye, or, at least, of communicating to an inflammation excited by other causes, such differences in character as often to render it difficult to recognize a disease with which we are well acquainted in its simple or idiopathic form.

"By the influence of local sympathy, inflammation of one texture of the eye never takes place, without extending in some degree to the textures with which the first affected is in contact; by the same influence an inflammatory disease originating in one texture of the eye shall be communicated to several of the other textures, the inflammation of the superficial tunics being communicated to those more deeply seated, and conversely that of the internal parts spreading outwards; and, while each texture obeys its own laws of morbid action, the whole organ in this way may become involved by what had at first a very limited existence, and perhaps a very trivial aspect." (P. 381.)

The term ophthalmia is indeed a very vague one, and of this every person must be convinced, from meeting with its use so constantly in the reports of various authors, without any definite meaning being conveyed to the mind of the reader. Thus, when Mr. Calvert (vide *Reflections on Fever*, 1815,) speaks of "cases of ophthalmia where 160 or 170 ounces of blood were said to have been taken (unsuccessfully, however,) in the space of three days," we are at a loss to know what particular form of ophthalmia it could have been to justify so dangerous a depletion. Our author very justly pursues the subject as follows.

"When we reflect, then, on the innumerable combinations which may take place among the inflammatory diseases of the eye, and the many causes by which these diseases may be modified, we shall be convinced, I think, that of all the subjects requiring descriptions and explanations of morbid actions and changes, there can be few more difficult than those diseases which have been swept together with so indiscriminating a hand under the name of *ophthalmia*. To consider these actions and changes individually, and only in a single texture of the eye at once, may seem to lessen the difficulty; for instance, to consider inflammation of the cornea, and to exhibit to ourselves in order, effusion of serum, effusion of coagulable lymph, secretion of pus, formation of abscess, ulceration, mortification, and cicatrization, according as each of these processes manifests itself in the cornea. But, to do all this, is to consider and to exhibit what never takes place separately in nature. Unless this be kept in mind by those who begin to study the inflammatory diseases of the eye, they will be not a little perplexed by the diversified complications of morbid phenomena which they will meet at every step of their progress.

"The knowledge of the inflammatory diseases of the eye has been greatly retarded by the practice of confounding them all under the name of ophthalmia, and thus overlooking both the seat of the disease, and the peculiar nature of the inflammation. The consequence of thus viewing all these diseases without discrimination, has been a method of treating them equally preposterous. In fact, in the practice of those who have had no opportunities of properly studying the diseases of the eye, one routine of remedies continues to be used in every case in which the eye appears inflamed; and it often happens that it is not till this routine is exhausted, and the eye, in some of its essential parts, becoming seriously disorganized, that a suspicion arises of there being something specific or peculiar in the case. Even from the slight view which we have already taken of this subject, it is evidently impossible that the inflammatory affections of parts so widely differing in structure and function as do those which are assembled in the eye, can be treated at once indiscriminately and successfully. We find, for example, that the remedies which in the course of a few days are sufficient completely to remove inflammation of the conjunctiva, only aggravate inflammation of the sclerotica or iris; while the plan of treatment which speedily cures sclerotitis or iritis, if trusted to in conjunctivitis, would expose the eye to almost certain destruction. Great advantages will accrue, then, from the adoption of an accurate classification of the ophthalmiæ. One advantage of no inconsiderable moment will be, that we shall conduct our examinations of the inflammatory diseases of the eye which may come under our care with much more accuracy than we could possibly do, were we to employ the vague nomenclature commonly used upon this subject. Having noted exactly the disease which is before us, we shall be able both to ascertain, to our own satisfaction, the effects of the remedies which we employ, and to communicate our experience to others, which, without a just classification and perspicuous nomenclature, it is utterly impossible to do." (P. 382.)

At the conclusion of this section we find a table of the varieties of inflammatory action in the different tissues of the eyeball, somewhat minutely divided as regards certain textures, and, as it appears to us, scarcely sufficiently discriminative in others. For instance, scrofulous conjunctivitis includes in this table all cases of phlyctenulæ and pustule. Now, this we think much too comprehensive, as there are undoubtedly cases in which these appearances exist without the scrofulous diathesis being present; and equally true is it, that the strumous ophthalmia, characterised by its most prominent symptom, intolerance of light, shall for months afflict the patient, without the remotest appearance of either.

In glancing over the history and symptoms of this disease, the scrofulous conjunctivitis, we find the following observations on the distressing photophobia almost invariably present during some part of its progress.

"The intolerance of light, in this disease, has by one author been regarded as depending on an affection of the retina, an idea which appears to derive some degree of support from the fact, that, in the dusk the patient is able to open his eyes, whereas were this symptom dependent merely on the state of the conjunctiva, it would remain the same in obscure as in bright light, and be more marked in catarrhal than in phlyctenular ophthalmia.

"In one case which I saw, the intolerance of light and spasm of the lids had continued for more than a year. When at length they abated, which they did of themselves, without the influence of medicine, (the mother having neglected to attend at the Eye Infirmary,) the child groped with its hands, as if blind, although it saw; so strongly confirmed was the habit of using the sense of touch in preference to that of sight.

"In another case, on the photophobia subsiding, we discovered the child to be amaurotic, although, until seized with the ophthalmia, it had seen perfectly." (P. 453.)

When a particular inflammatory disease affects one membrane of the eye, this, if unchecked, and of sufficient violence, will often gradually proceed to the implication of contiguous tissues, until the whole organ becomes involved in one general action; thus, the phlyctenula may give birth to a penetrating ulcer of the cornea; the aqueous fluid now escapes, the iris prolapses, and general inflammation of the anterior chamber ensues. This may go on till the internal tunics are attacked, and the retina, in its turn, suffers, and becomes amaurotic. But that the intolerance of light, which forms one of the most distressing symptoms, and sometimes almost, we might say, the only one, is dependent upon an "affection [inflammation] of the retina," we cannot bring ourselves to allow. Considering the delicacy of this membrane, and the permanent impairment of vision which frequently follows acute or chronic inflammation of its tissue, we confess that the rarity of amaurosis, and even of defective vision, after strumous ophthalmia, (unless when it arises from mechanical obstruction to the rays of light, from opacities, &c.) seems to contradict such an hypothesis.

The following is a very good specimen of the disease in its aggravated form; and is here quoted, to show the phenomena attendant upon scrofulous ophthalmia, and that the obstacle to vision was rather from mischief done to the anterior chamber, than from any participation of the retina in the inflammatory action, although the intolerance of light was excessive.

"CASE II. James Tassie, aged eight, was admitted on the 15th of August, 1828, with phlyctenular ophthalmia of the right eye. He had been troubled with this complaint, more or less, for seven years. There was formerly a considerable albugo on the right cornea, but it had diminished much till within a fortnight before his admission, when a relapse took place. The cornea appeared to be rough and nebulous, but the intolerance of light was so great that it was with difficulty that any part of it could be exposed. The nitras argenti solution was applied, and he had a solution of tartar emetic, in divided doses, till vomiting was produced. Next day he could open the eye better; and an onyx was now observed at the lower edge of the cornea, which had not been perceived on the previous day. He was ordered to take a grain of sulphate of quina thrice a day, and to use the murias hydrargyri collyrium. By the 18th the onyx was gone. The extract of belladonna was applied to the eyebrow and forehead, some fears being entertained regarding the state of the iris. By the 20th, the intolerance of light having considerably subsided, the cornea could be more completely seen. The centre of it was found to be perforated by an ulcer, and the pupil contracted. On the 22d, the eye continued easier, but the iris was observed to be everywhere in contact with the cornea. The sulphate of quina, belladonna, and collyrium, were continued. On the 27th, the iris appeared to be returning a little into its natural place, the pupil was pretty visible, and he saw a little with the eye. On the 28th, the pupil was evidently expanding, and the cornea clearing. By the 1st of September, the pupil was free of the cornea, except at its inner edge, where it still adhered by a single point. By the 16th, the iris was entirely free. Soon after this the ulcer of the cornea cicatrized, the speck gradually cleared, and the eye retained a very considerable share of vision." (P. 461.)

There is no question that, during the existence of this disease, the sensibility of the retina is increased to a morbid degree of exquisite suffering, and we agree with Mr. Lawrence, (vide *Treatise on Diseases of the Eye*, p. 246,) that this is not so dependent upon vascular action "as upon irritability of the structure, and a sympathetic or functional affection." It becomes a matter of much importance to put this question on a proper footing, since, in our prognosis, we are hereby enabled to assure the friends of the patient that one of the most grievous symptoms, and which to them appears so alarming, does not involve risk to the sight, permanent or even present. Of this indeed we may assure ourselves, by seeing that the little patient, after sitting all day with its head buried in the bosom of its mother, hiding from the light, and screaming with pain on the least attempt to uncover the eyes, will, on the approach of twilight, open its lids, and observe objects with distinctness and freedom from pain. This certainly could not be done were the retina actually inflamed.

In the table of Ophthalmiæ, at p. 383, we find No. 3, "Leucorrheal or Ophthalmia Neonatorum," and at p. 432 a description of this disease. The symptoms of the purulent ophthalmia of infants are too well known to make it necessary to quote Dr. Mackenzie's very excellent description. We may say, however, that, although there can be no doubt but that the mother's being affected with leucorrhœa at the time of birth is a very common cause, yet it is not so uniformly present as to justify us in adopting the term leucorrhœal as the generic name of this disease.

After having detailed the general symptoms, our author proceeds to deny the opinion of Mr. Saunders, that the destruction of the cornea is produced by the process of sloughing, thus—

"His opinion regarding the mode in which the cornea is destroyed in this disease appears of more importance, but equally incorrect. He maintains that it is by sloughing, not by suppuration and ulceration, that the destruction of the cornea is effected. The opportunities which I have had of watching the progress of the affection of the cornea have convinced me of the contrary. Onyx or infiltration of pus into the substance of the cornea, is the uniform harbinger of destruction: the lamellæ exterior to the pus give way by ulceration; the ulcer spreads and deepens, till the cornea is penetrated, and often almost quite destroyed. Any thing like mortification, or sloughing, I have never seen. The coming away of the purulent infiltration, exposed by ulceration, must have given rise to Mr. Saunders's notion of successive sloughs." (P. 433.)

Now, that onyx and infiltration of pus between the laminæ of the cornea does not unfrequently take place, we have no wish to deny; yet we have undoubtedly seen, in cases where the disease has been neglected, sloughing of the cornea, at times implicating the whole substance of this tunic. Even before opening the eye of the infant, we are sometimes certain, from the purple lividness of the swollen lids, that the cornea is in a state of mortification, or that the slough has either wholly, or in part, detached. In some instances we have seen the cornea, of a dirty grey or brown colour, separate from its circumference, and fall out as a watchglass from its verge, followed, in one particular instance we witnessed, by the lens, which had to a considerable extent lost its original transparency.

The prognosis of this disease is accurately and tersely expressed.

"*Prognosis.* If the corneæ are only free from ulceration, and from purulent infiltration, how violent so ever the inflammation may be and profuse the discharge, our prognosis is favorable, the sight is safe. If the disease has been allowed fairly to establish itself, and its progress not interfered with for eight days or longer, it often proves tedious; six, eight, or ten weeks elapsing before it is perfectly cured. It is always more difficult to overcome, when the child is exposed to cold damp air, ill nourished, improperly fed, or when the nurse drinks spirits or porter. If there is superficial ulceration, without onyx, probably a slight speck may remain. If the ulceration is deep, an indelible opacity must be the consequence. If the iris is protruding through a small penetrating ulcer, the pupil will be permanently disfigured, and vision more or less impeded. If the ulcer is directly over the pupil, the probability is that the pupillary edge of the iris will adhere to the cicatrice, and vision be lost until an artificial pupil be formed in after-life by an operation. If there is a considerable onyx, we can promise nothing; for although, under proper treatment, the matter may be absorbed, this is by no means a certain result: the purulent exudation may, on the contrary, increase, the cornea burst, and the eye become partially or totally staphylomatous. Whenever the person who brings the child to me announces that the disease has continued for three weeks or longer, I open the lids of the infant with the fearful presentiment that vision is lost, and but too often I find one or both of the corneæ gone, and the iris and humours protruding. In this case, it is our painful duty to say that there is no hope of sight." (P. 434.)

The treatment is also ably detailed; but the simple remedy which we have seen so successful in many hundred cases is not even mentioned by name: we mean the solution of alum, (gr. iv. vel x. ad ℥j. Aq. destillat.) As in the majority of instances the application of the means employed, especially with the poorer classes, must necessarily be left to the mother or the nurse, we are unwilling to trust the nitrate of silver, or other active stimulants and astringents, to their hands; while, in prescribing the solution of alum, we feel perfectly satisfied as to the result: we have the satisfaction of knowing also that the application is not only perfectly safe, but that it can scarcely be abused.

In many hundred instances where this mode of treatment has been adopted, we hardly remember one in which it has failed to produce a perfect cure. We of course speak of those cases only which are brought to the surgeon prior to infiltration, onyx, or ulcer.

When, however, the disease has proceeded to the extent of endangering the cornea, the mode of treatment certainly becomes more complicated, and we would rather trust to the application of a single leech, with the sulphate of quinine, as recommended at rule 9, p. 437, than to the use of calomel, which, employed indeed at any stage of the disease, would, we fear, hasten the destructive ulceration of this membrane.

We may observe, in passing, that cataract is but a rare result of purulent ophthalmia unaccompanied by synechia; since, fortunately, unless the cornea be penetrated by ulceration, the internal structures seem but little affected in this disease.

Accuracy of diagnosis is of the highest importance in all diseases, but more especially so in the eye, where the safety of the organ often depends upon our first decision as to the specific nature of the affection; we are therefore grateful to all who help us to those external characters which, however trifling, taken separately, by their combination unravel some of the hidden mysteries of morbid action. We have thus, at p. 395, the objective and subjective symptoms of the varieties of ophthalmia. To this are attached four diagrams explanatory of the text, which the descriptions quoted will sufficiently indicate.

" Either by looking at the inflamed eye, and particularly by observing the arrangement of the enlarged blood-vessels, without hearing the patient's account of his sensations, or by learning from the patient the kind of pain with which he is affected, without looking at the eye, a tolerably correct notion may in general be formed of the kind of ophthalmia which is present. Of course, before proceeding to treat any particular case, we avail ourselves of all the symptoms, both objective and subjective, both what are offered to the direct examination of our own senses, and what we must receive on the testimony of the patient.

" § 1. *Arrangements of the Blood-vessels.* We meet with four arrangements of the external vessels, in the ophthalmiæ; namely, the reticular, the zonular, the fascicular, and the varicose.

" 1. The network observed in the first of these arrangements is seated in the conjunctiva, the vessels of which it is formed are comparatively large, and tortuous, they anastomose freely with one another, and can be shoved or drawn aside, by pressing or dragging the eyelids with the finger. This arrangement is characteristic of puro-mucous conjunctivitis.

" 2. In zonular inflammation, the vessels are small and hair-like, never very tortuous, but running like separate radii towards the cornea; thus forming, not a network, but a halo, over which the conjunctiva is easily made to slide. This arrangement belongs to sclerotitis and iritis.

" 3. In the preceding arrangements, the enlarged vessels are spread pretty equally over the eyeball; but in the fascicular, the redness commonly occupies only one side of the eye, and often consists of a few vessels only, running towards the cornea, and terminating in a phlyctenula or pustule. This arrangement, then, belongs to the scrofulous varieties of conjunctivitis.

" 4. Large tortuous vessels, derived from those belonging to the recti muscles, constitute the varicose arrangement, which we meet with most frequently in the chronic stage of arthritic ophthalmia, and in choroditis. The vessels in question are branches of one or other of the seven trunks, which, advancing towards the cornea, are visible in every eye; namely, one from the rectus externus, and two from each of the other recti.

"These four arrangements of vessels are, in general, perfectly distinct; but, in some cases, they are mixed together, or are obscured by what is termed chemosis, that is, an inflammatory œdema of the cellular substance under the conjunctiva, so that this membrane is raised from the sclerotica, and so much swollen as sometimes to overlap the edge of the cornea, or even protrude from between the eyelids. When chemosis is present, nothing can be seen of the particular distribution of the vessels. In the compound ophthalmiæ, again, such as the catarrho-rheumatic, pustulo-catarrhal, &c. two or more of the arrangements may be combined.

"§ 1. *Kinds of Pain.* Two different varieties of pain attend the ophthalmiæ, the one being characteristic of the inflammation of the conjunctiva, the other of those affecting the sclerotica and iris. The former is uniformly compared by the patient to the feeling which is produced by sand in the eyes; it is most felt during the day, and especially in the morning, when the eyes begin to be moved; the latter is pulsatory, affects the circumorbital region as much as the eye itself, and is strikingly nocturnal, commencing after sunset, increasing in violence till after midnight, and abating toward sunrise, scarcely felt during the day, but returning about the same hour in the evening. Ophthalmiæ attended by the conjunctival or sandy pain, are generally curable by external applications; those which are accompanied by the circumorbital or pulsatory pain, always require venesection." (P. 395.)

We are afraid we have not left ourselves much room for further remarks, although we wish we had it in our power to notice more fully the very many excellent cases with which the work teems.

Mr. Lawrence, near the end of his treatise, introduced the report of a case of entozoa found in the anterior chamber of the eye of a patient in the Glasgow Ophthalmic Infirmary: the result, however, was not known when his book went to press; we shall therefore not apologize to our readers for quoting the whole history, with its termination, expressing our regret, however, that the animal (*Cysticercus cellulosæ*) was not extracted entire or alive, in order that it might have been subjected to microscopical examination.

"Case. From the month of August 1832, till about the middle of January 1833, when she was first brought to Mr. Logan, the child had suffered repeated attacks of inflammation in the left eye. Mr. L. found the cornea so nebulous, and the ophthalmia so severe, that he dreaded a total loss of sight. He treated the case as one of scrofulous ophthalmia; and, after the use of alterative medicines, and the application of a blister behind the ear, the inflammatory symptoms subsided, leaving, however, a slight opacity of the lower part of the cornea. After a week, the child was again brought to Mr. L. who, on examining the eye, discovered, to his great surprise, a semitransparent body, of about two lines in diameter, floating unattached in the anterior chamber. This body appeared almost perfectly spherical, except that there proceeded from its lower edge a slender process, of a white colour, with a slightly bulbous extremity, not unlike the proboscis of a common fly. This process, Mr. L. observed to be of greater specific gravity than the spherical or cystic portion, so that it always turned into the most depending position. He also remarked that it was projected or elongated from time to time, and again retracted, so as to be completely hid within the cystic portion; while this, in its turn, assumed various changes of form, explicable only on the supposition of the whole constituting a living hydatid.

" On the 3d April, when I examined the case, I found the cornea slightly nebulous, the eye free from inflammation aud pain, and the appearances and movements of the animal exactly such as described by Mr. Logan.

" When the patient kept her head at rest, as she sat before me, in a moderate light, the animal covered the two lower thirds of the pupil. Watching it carefully, its cystic portion was seen to become more or less spherical, and then to assume a flattened form, while its head I saw at one moment thrust suddenly down to the bottom of the anterior chamber, and at the next drawn up so completely as scarcely to be visible. Mr. Meikle turned the child's head gently back, and instantly the hydatid revolved through the aqueous humour, so that the head fell to the upper edge of the cornea, now become the more depending part. On the child again leaning forwards, it settled like a little balloon in its former position, preventing the patient from seeing objects directly before her, or below the level of the eye, but permitting the vision of such as were placed above.

" Mr. Logan had observed no increase of size in the animal while it was under his inspection. Mr. Meikle had watched it carefully for three weeks, without observing any other change than a slight increase in the opacity of the cystic portion.

" To every one who had seen or heard of Mr. Logan's case, the question naturally occurred, ought not this animal to be removed from the eye? Mr. Logan and Mr. Meikle appeared to have deferred employing any means for destroying or removing it; first, because it seemed to be producing no mischief; and, secondly, because there was a probability that it was a short-lived animal, and likely therefore speedily to perish, and shrink away, so as to give no greater irritation than a shred of lenticular capsule. Various means naturally suggested themselves for killing the animal; such as passing electric or galvanic shocks through the eye, rubbing-in oil of turpentine round the orbital region, giving this medicine internally in small doses, or putting the child on a course of sulphate of quina, or of some other vegetable bitter known to be inimical to the life of the entozoa. As the patient appeared to be in perfect health, it was natural to suppose that the other organs were free from hydatids, and that a change of diet would have little or no effect upon the solitary individual in the aqueous humour. Had she, on the contrary, presented a cachectic constitution, with pale complexion, tumid belly, debility, and fever, none of which symptoms were present, we should have been led to suspect that what was visible in the eye was but a sample of innumerable hydatids in the internal parts of the body, and might have recommended a change of diet, with some hopes of success.

"In the course of six weeks after I saw the patient, the cysticercus having enlarged in size, the vessels of the conjunctiva and sclerotica become turgid, the iris changed in colour, and less free in its motions, while the child complained much of pain in the eye; it was decided that the operation of extraction should be attempted, and I owe to Dr. Robertson, of Edinburgh, who operated, the communication of the following particulars.

"The incision of the cornea was performed without the slightest difficulty, but no persuasion or threats could induce the child again to open the eye; she became perfectly unruly, and the muscles compressed the eyeball so powerfully, that the lens was forced out, and the hydatid ruptured. The patient was put to bed in this state. In the evening, Dr. R. succeeded in getting the girl to open the eyelids, when with the forceps he extracted from the lips of the incision the remains of the animal in shreds, it being so delicate as scarcely to bear the slightest touch. A portion of the iris remained in the wound, which nothing would induce the girl to allow Dr. R. to attempt to return.

"After the eye healed the cornea remained clear, except at the cicatrice, where it was only semitransparent; the pupil, in consequence of adhesion to the cicatrice, was elliptical, and the opaque capsule of the lens occupied the pupillary aperture. The patient readily recognised the presence of light." (P. 967.)

Two engravings accompany this case, and are useful in explaining the appearances described.

We are led, in conclusion, to say a few words relative to the plates in general which illustrate this edition of Dr. Mackenzie's book. We are at all times adverse to the introduction of engravings into the body of the type, which are necessarily, we believe, either woodcuts or casts in metal from the block. Now, although we are aware the plates to this work are intended simply as diagrams, yet woodcuts are so particularly unsuited to the very nature of the diseases intended to be represented, that we almost doubt that they are disadvantageous to the student, as leading to an erroneous or very imperfect impression of the morbid appearances of the organ. In fact, if there be any class of diseases which more than another requires accuracy of drawing and delicacy of touch, it is the morbid conditions of the organ of vision. Not only should the engravings be of the highest order of art, but, where so much depends upon variations of tint, and degrees of opacity or transparency, they ought to be coloured with fidelity, and by one accustomed to such pursuits. We are well aware that such plates would add most materially to the expense of the book, and put it beyond the reach of students in general; but Dr. Mackenzie's work is not for such readers alone; nor do we know any medical treatise in the English language which better deserves to be accompanied by an atlas of engravings of the character we have described: such we yet hope to see in a future edition of this admirable book. The frontispiece, indeed, forms an honourable exception to the criticism we have ventured to apply to the rest of the plates.

